# Improvement of wear resistance in a pearlitic rail steel via quenching and partitioning processing

**DOI:** 10.1038/s41598-019-43623-7

**Published:** 2019-05-15

**Authors:** Mohammad Masoumi, Edwan Anderson Ariza Echeverri, André Paulo Tschiptschin, Hélio Goldenstein

**Affiliations:** 1 Centro de Engenharia, Modelagem e Ciências Sociais Aplicadas, Universidade Federal do ABC, 09210-580, Santo André, SP Brazil; 2 Departamento de Engenharia Metalúrgica e de Materiais, Escola Politécnica da Universidade de São Paulo, 05508-030 São Paulo, SP Brazil; 3 Escuela de Tecnología Mecánica, Universidad Tecnológica de Pereira, Carrera 27 #10-02 Alamos, Pereira, Risaralda, Colombia

**Keywords:** Materials for devices, Mechanical engineering

## Abstract

Improvement of wear resistance and mechanical performance of rails used in heavy-haul railway are essential to reduce railroad maintenance costs. A novel heat treatment based on quenching and partitioning (Q&P) processing was proposed to improve the wear resistance of a hypereutectoid pearlitic rail. 50% of austenite was transformed into martensite under an interrupted quenching from full austenitization temperature to 140 °C. A multiphase microstructure resulted from the quenching and partitioning process, consisting of tempered martensite, bainite, retained austenite, and pearlite colonies. The partitioning step was performed in the range of 350–650 °C. Microstructure characteristics were investigated using scanning electron microscopy, microhardness measurements, X-ray and electron backscattered diffraction. Uniaxial tensile and pin-on-disc tests were also performed to evaluate the mechanical properties and wear resistance. The best combination of wear resistance and mechanical performance was obtained in samples partitioned at 450 and 550 °C, which may be applied in the railway industries.

## Introduction

Pearlitic rail steel is still widely used in heavy-haul railway all around the world, because it does not contain any major alloying elements, presents a low production cost and it has relatively good mechanical properties and wear resistance^1–4^. Rails life is strongly impacted by wear, including the abrasive, adhesive, impact by erosion-percussion and fatigue mechanisms^5^. To increase rail service life and prevent catastrophic accidents, railway companies spend millions of dollars annually grinding to remove surface cracks and spalls, caused by rolling contact fatigue. The wear resistance of pearlitic rails is improved through enhancements in steel cleanness, by the development of head-hardened rail, by hardness and carbon content increase, and by refining pearlite spacing^2–4^.

Head-hardened rails exhibit superior wear behavior and rolling contact fatigue resistance due to the refining of the pearlitic structure caused by rapid cooling^6^. They are produced by reheating the head of rails using induction heating, followed by accelerated cooling with a mixture of air and water. The yield strength of 1200 MPa can be achieved in the rail-head by this process^4^. Moreover, controlling the concentration of alloying elements (i.e., carbon, manganese, silicon, chromium, vanadium, etc.) is essential for improving the wear resistance, as they play an essential role in determining the microstructure and hence the mechanical performance. Carbon increases the volume percentage of hard cementite, manganese, and chromium raises the hardness and the strength of the pearlite by slowing the kinetics of the eutectoid transformation, which on continuous cooling translate into lower transformation temperatures and by solid solution strengthening of both ferrite and cementite phases, silicon provides solid solution strengthening in ferrite phase, and vanadium improves hardness and the steel strength by refining the prior austenite grain size and also by precipitation hardening by formation of vanadium carbides and carbonitrides^7–9^.

In many tribological systems wear resistance significantly increases with increasing hardness, leading to the development of high strength rails with tempered martensite or bainite microstructures^10^. Quenching and tempering (QT) is a widely used heat-treatment, where the steel is transformed essentially to martensite, during a quenching step, followed by a tempering step, where martensite decomposes to ferrite and transition carbides, at low temperatures, or cementite above 350 °C. For high carbon steels, during the quenching step to room temperature, a small fraction of the parent austenite is retained, as the martensite finish (M_F_) temperatures is below room temperature. Recently, Speer *et al*.^11^ introduced a novel approach of treatment called the quenching and partitioning (Q&P) heat treatment. Q&P treatment is a two steps process: initially, an interrupted quenching from full austenitization to a temperature between the martensite start (M_S_) and M_F_ temperatures, resulting in partially transformed structure containing retained austenite. The second step is a partitioning treatment at a relative high temperature to enhance carbon diffusion from supersaturated martensite into neighboring untransformed austenite, stabilizing it at room temperature. The possibility of stabilizing austenite by controlling the carbon enrichment, during the partitioning step, differentiates the Q&P from the traditional QT process. Additionally, superior mechanical properties of Q&P steels have been obtained in comparison to steels with conventional heat treatment/microstructures such as bainitic or martensitic microstructures^10,12^.

Multiphase microstructure, consisting of tempered martensite, bainite, stabilized austenite, sometimes mixed with secondary martensite, known as MA microconstituent, and even pearlite colonies, is developed during Q&P process. The eutectoid colonies developed during a Q&P heat treatment of hypereutectoid steel^10^ show higher hardness than conventional pearlite. Therefore, it is expected for the material to exhibit superior strength and wear resistance, which is highly sought properties in rails for heavy haul railways. In the current work the effects of Q&P heat treatment on the microstructure, tensile properties, and wear resistance of a conventional hypereutectoid rail steel were investigated, aiming to improve wear resistance and ultimately minimize maintenance cost/time of heavy haul railways.

In this work, short soaking times for both quenching and partitioning stages, 10 s and 30 s, respectively, were employed in order to minimize or prevent carbide precipitation, as commercial grade rail steels, with low Si and residual Al contents, were used. Although literature provides evidence that Si and or Al prevent or at least retard cementite precipitation, Kim *et al*.^13^ and Ariza *et al*.^14^ reported that even with high concentrations of Si (as high as 1.98 wt%), the formation of cementite was still observed. Toji *et al*.^15^ also observed cementite precipitation in a high carbon steel, even though 2 wt. % Si was added to the chemical composition to prevent cementite formation. Allain *et al*.^16^ studied the kinetics of carbon precipitation during the Q&P process using *in situ* High Energy X-Ray Diffraction and Transmission Electron Microscopy (TEM) techniques. They pointed out that at least 200 s are required at 400 °C for carbon redistribution, austenite reversion, and the formation of carbides. Therefore, Si and/or Al are added to suppress cementite precipitation during long partitioning periods in Q&P steels^16^. Ariza *et al*.^17^ suggested that the isothermal holding time in Q&P processes need to be kept short to avoid the precipitation of isothermal transformation products. Recently, Bansal *et al*.^18^ studied the microstructure evolution of a Al-free 0.5 wt.% Si steel through Q&P process. They confirmed the presence of retained austenite (thin film and blocky morphologies), during partitioning at 330 °C for 2 min, and no cementite precipitation was observed. The authors state that there are studies available on Q&P treatment in steels with higher Si and/or Al additions (Si + Al = 1 to 1.7 wt.%), but the effect of low Si contents and no Al additions, has not been given due consideration^18,19^.

## Material and Methods

**Figure 1:**
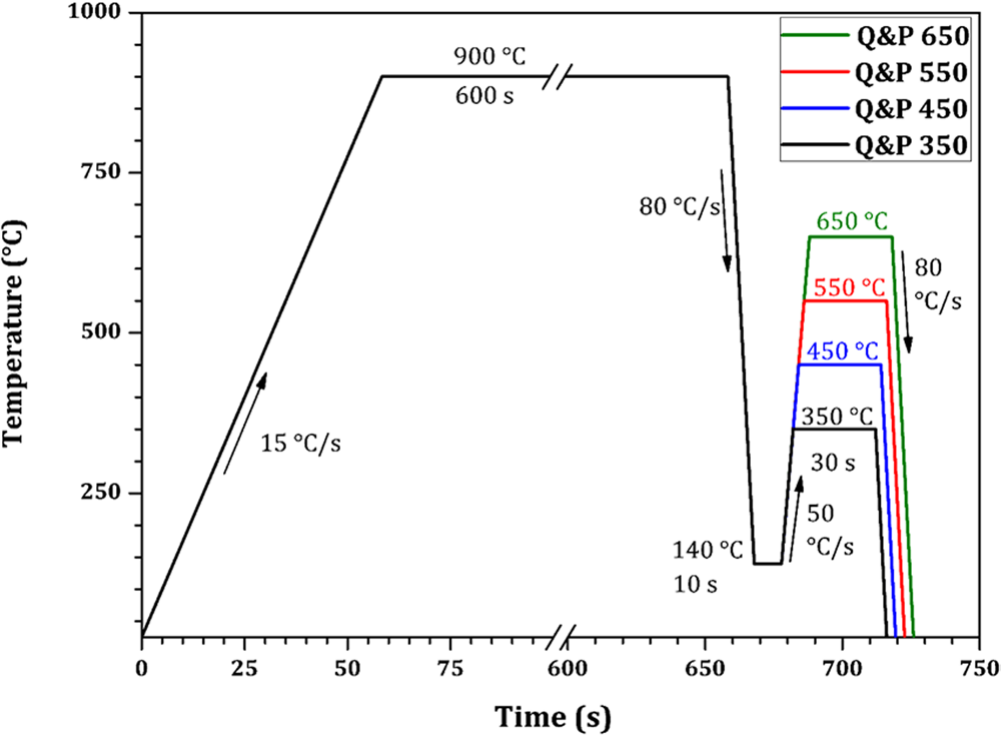
Schematic representation of the different quenching and partitioning treatment schedules (time vs temperature).

**Figure 2:**
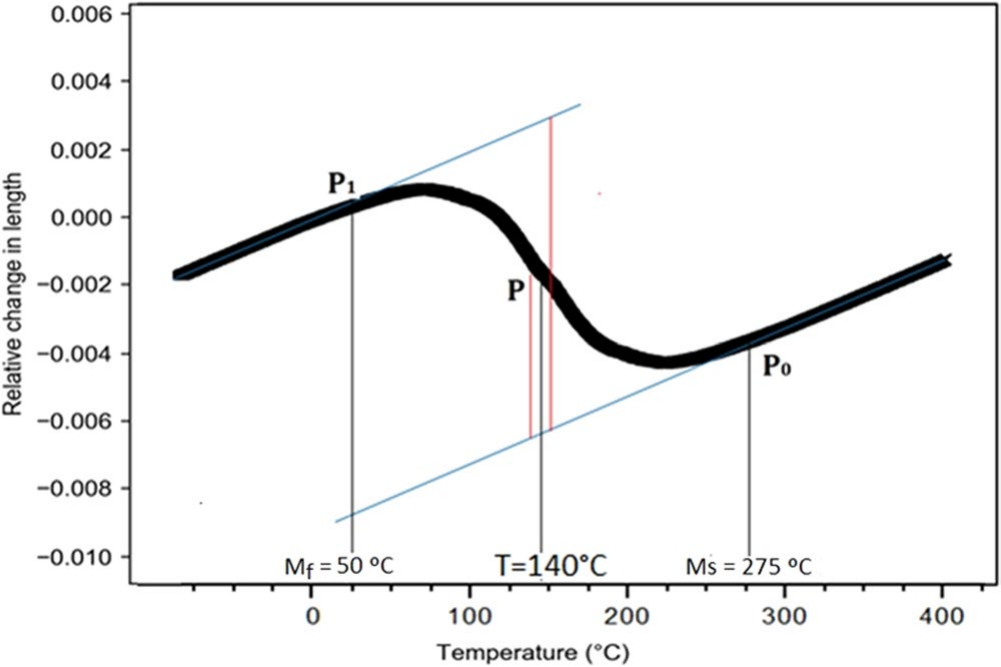
The relative length change with temperature of dilatometric measurements in the region of the martensitic transformation.

A standard carbon grade rail steel with chemical composition Fe- 0.75 wt% C- 1.03 wt% Mn- 0.22 wt% Si- 0.21 wt% Cr was investigated in this research. Quenching and partitioning processing of the samples was performed in a DIL805 dilatometer produced by BÄHR-Thermoanalyse GmbH. Cylindrical specimens with 4 mm in diameter and 10 mm height were cut from the bottom of the railhead, which was previously subjected to head hardening processing to increase wear resistance. Specimens were initially austenitized at 900 °C for 600 s and then rapidly cooled to 140 °C in helium gas, temperature where 50% of austenite is transformed into martensite. Partitioning process was performed at 350, 450, 550, and 650 °C for 30 s. The schematic representation of the different quenching and partitioning treatment schedules is shown in Fig. 1. Initially, dilatometry experiments were performed to determine the martensitic start (Ms) and finish temperatures (Mf). Ms and Mf were determined as 275 °C and 50 °C, respectively. The martensite fraction was calculated by Equation (1) from diagram of length change in the region of martensitic transformation, obtained from dilatometric data^20^, Fig. 2;

1 $$f(T)=(P(T)-{P}_{0}(T))/({P}_{1}(T)-{P}_{0}(T))$$

where *f* is the fraction of martensite, and T is the temperature to which the material is quenched.

The microstructure of the specimens was examined using scanning electron microscopy (SEM, FEI-Inspect F50). Metallographic samples were prepared by mechanical grinding using SiC paper up #1200, followed by polishing with diamond paste (6, 3, and 1 µm). The microstructure was revealed by etching in 5% Nital for approximately 30 s. The distinction between ferrite and Fe_3_C by secondary electrons in SEM imaging is facilitated by the use of deep etching techniques. Thereby, higher etching times were used in this work. Morphological details of microstructural constituents were observed with secondary electron images of SEM with accelerating voltage of 20 kV and working distance of 10 mm. Moreover, the microhardness was measured using a Shimadzu (HMV-2TADW) microhardness tester with 2.97 N (300 g) of load for 15 s (HV_0.3_). The reported microhardness values are the average from 10 indentations.

X-ray diffraction was carried out on un-etched samples, after polishing to 1 μm, using an X-pert PRO diffractometer with filtered Cu*K*_*α*_ radiation. A continuous scanning mode was made over the angular width 2θ = 30–100°, with 0.02° angle step and collecting time of 2 s at each step. The mentioned Bragg angle range covered the {111}, {200}, {220} and {311} peaks of retained austenite and {110}, {200}, {211} and {220} peaks of martensite. Finally, the fraction of retained austenite was estimated using Rietveld refinement^21^ by X’Pert HighScore and GSAS + EXPGUI software^22^.

Electron Backscatter Diffraction (EBSD) measurements were done to study the crystallographic features of the dilatometric samples. The equipment used was a FEI-Inspect F50 SEM equipped with a field emission gun (FEG) and EBSD detector. Before the EBSD measurements, the samples were prepared according to the standard preparation and polished with 50 nm colloidal silica slurry for 3 h. All analyses were carried out with accelerating voltage of 20 kV, spot size of 5, working distance of about 12 mm, and step size of 20 nm with hexagonal scan grid mode. The EBSD and orientation data were analyzed using TSL OIM data analysis 7 and MTEX – Free and Open Source Software Toolbox.

Pin-on-disc tribological test is widely used in several transportation fields (such as trains and road vehicles) to simulate and evaluate the wear performance of metallic materials^23,24^. The tribotest was carried out using pin-on-disk sliding friction test without lubrication, following ASTM G99-05 standard with five repetitions for each test. The treated pins with 4 mm in diameter and 100 mm in height were fixed, and slided against a disc counter body of quenched and tempered AISI H13 tool steel (with 600 HV), rotating at 40 rpm, using a dead weight (100 N) to provide a desired nominal contact pressure on the pin^25^. Tests were performed at room temperature on a disc with radius of 25 mm and constant sliding speed of 0.2 m/s, resulting in a sliding distance of 720 m. The friction force was monitored during the test, and the friction coefficient was calculated as the ratio between the friction force and the normal load. Moreover, the mass loss of the pin was determined on a scale with precision of 10^−5^ g. In addition, room temperature uniaxial tensile tests were performed using a Instron model 3369 testing machine. The subsize specimens were machined from the samples treated in dilatometer by wire cut erosion discharge machine, according to the De Knijf *et al*.^26^ paper. Strain evaluation was measured using a video camera during the tensile test. Engineering stress-strain data was reported from the average of three tensile samples at a strain rate of 2 × 10^−3^ s^−1^.

## Results and Discussion

**Figure 3:**
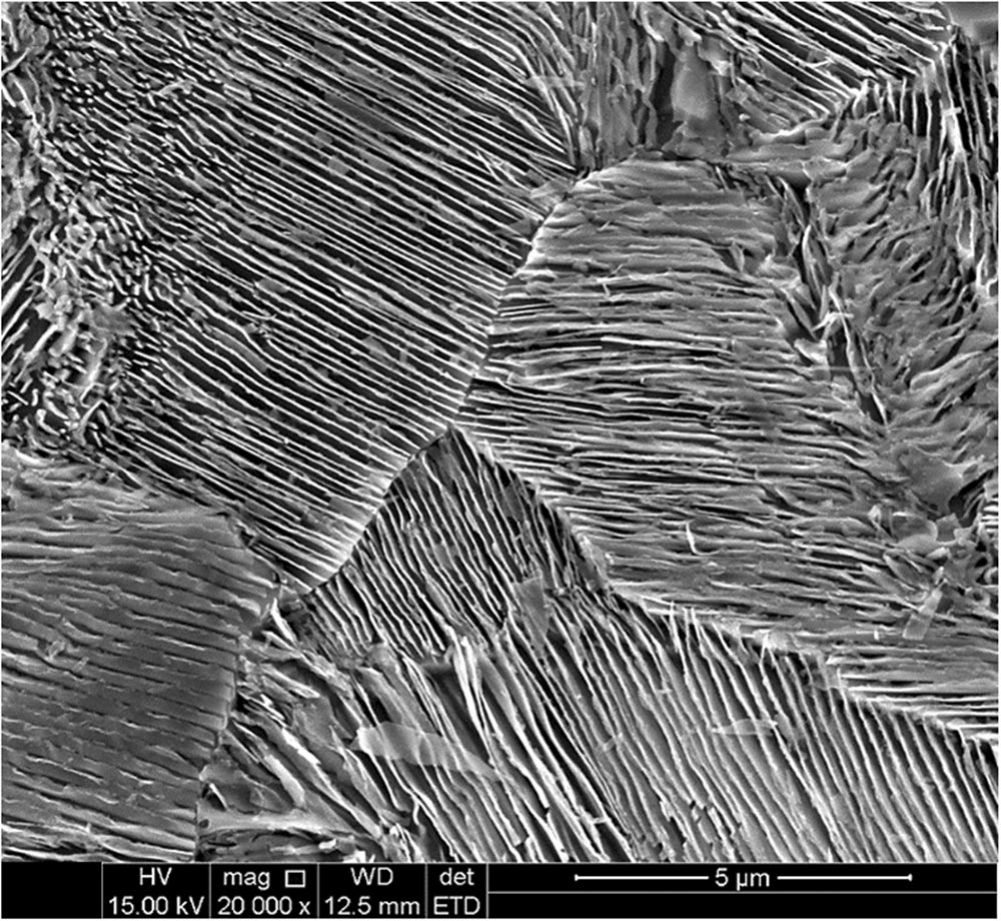
Secondary electron image of the initial microstructure of rail sample.

The initial microstructure of the investigated rail sample was fully pearlitic, as shown in Fig. 3. It can also be observed the random orientation of ferrite/cementite alternating lamellae colonies. The interlamellar spacing and the pearlite colony size, which are governed by the cooling rate^1^, control the mechanical properties and wear resistance of pearlitic steels. Using the linear intercept method^27^ and circular line method^28^, the average sizes of pearlite colony and the interlamellar spacing were approximately 15 µm and 0.25 µm, respectively. Proper mechanical properties and wear resistance are expected from the fine and fully pearlitic structure with low interlamellar spacing.

**Figure 4:**
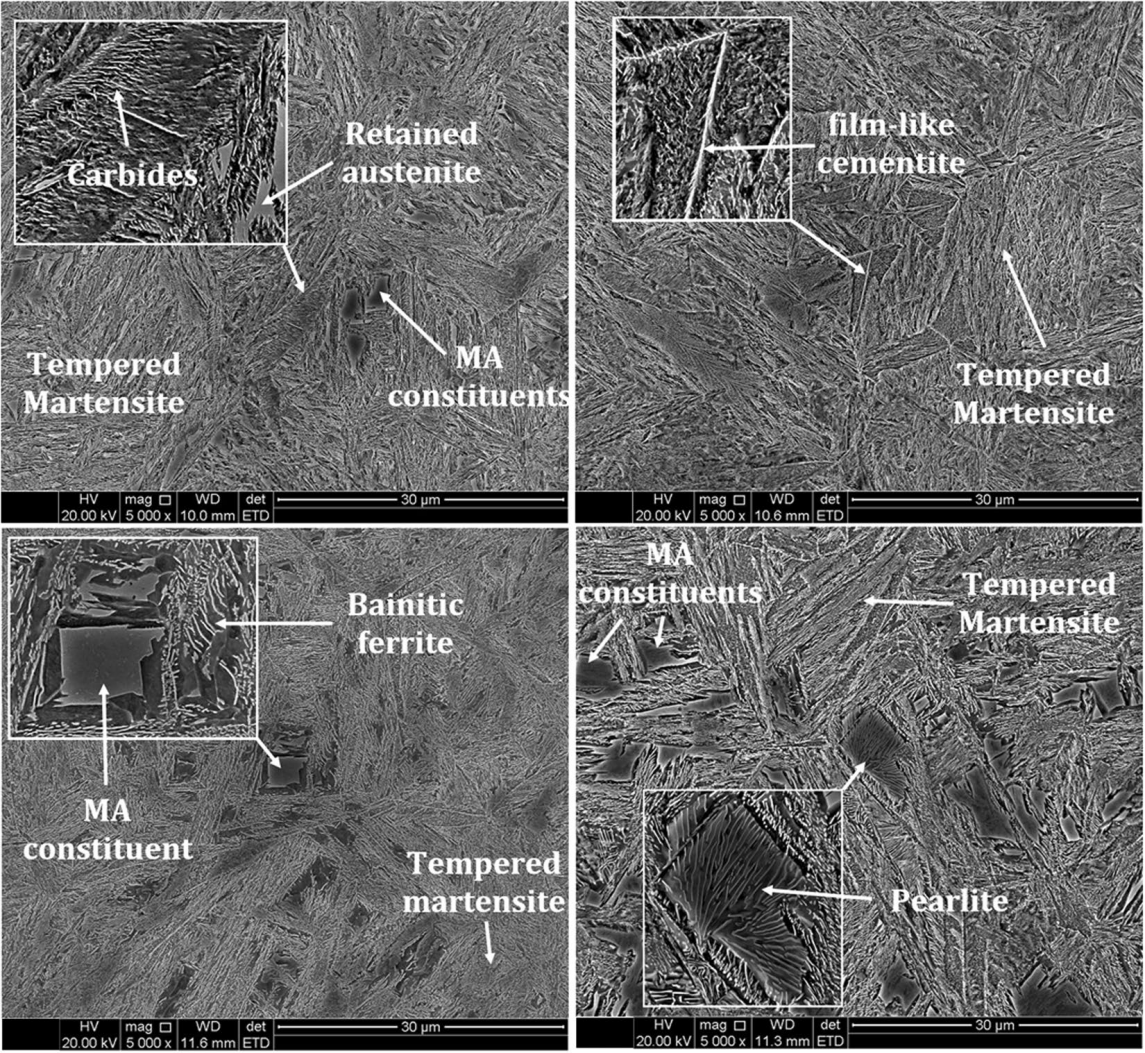
SEM images of the: (**a**) Q&P 350, (**b**) Q&P 450, (**c**) Q&P 550, and (**d**) Q&P 650 samples ref. to Fig. 1.

Figure 4 shows SEM images of quenched and partitioned samples at different temperatures. In the first quenching, at 140 °C, it is expected to form 50% martensite and 50% austenite. Then, partitioning at different temperatures led to the decomposition of retained austenite and the formation of various microstructures composed of martensite, bainite, pearlite, and fresh martensite. The Q&P 350 sample showed the martensitic microstructure. Also, the formation of carbides within martensite laths and fresh (untempered) martensite are observed in this sample. The average hardness was 615 ± 15 HV in this sample. Carbon atoms are rejected from martensite during the partitioning stage, since it is a carbon-supersaturated phase. During the partition step, a fraction of the original martensite carbon atoms precipitate as carbides (Fig. 4a); while the remaining fraction could partition to the retained austenite. Indeed, Toji^15^ reports experimental results, obtained by FE-EPMA and APT and proposed a model for the partition of carbon from martensite to austenite, in high carbon alloys, when simultaneous carbide precipitation occurs inside martensite. Carbon partitioning from martensite into austenite was clearly observed in the studied steels, even though considerable carbide precipitation was observed inside the martensite. A modified prediction model for the austenite carbon concentration after the partitioning step in the Q&P process, which can be applied to the case where carbide precipitation occurs in martensite, was proposed to explain the experimental results. At the second quenching, after partitioning, austenite partially transforms leading to the formation of MA constituent, containing fresh martensite, probably with a higher trapped carbon content, when compared to the nominal carbon content. Unetched fresh martensite formed at the final quenching is easily distinguished from etched martensite matrix. Gaude-Fugarolas^29^ reported that the remaining austenite is fully decomposed to bainitic-ferrite between 305 and 345 °C in moderate and high carbon content. Thus, the excess in carbon content enhanced the formation of transition carbide precipitates at lath martensite^30^.

Most recent work on Q&P shows that the partitioning of C is very fast, of the order of seconds or at most a few minutes^31^. As an example, Edmonds *et al*.^32^ studying carbon partitioning from martensite to austenite, using computational kinetic simulations and transmission electron microscopy observations for a 0.19%C-1.59%Mn-1.63%Si steel, reported that the martensite is depleted in carbon in less than 0.1 s. However, about 10 s are needed to homogenize the carbon profile inside the austenite at 400 °C^33^ or 375 °C^34^. The high C and low Si content of the commercial rail steel, allowing for competition between carbide precipitation and C partitioning to the austenite, prevented complete stabilization of the austenite, leading to the presence of MA constituent containing secondary martensite plates embedded in retained austenite blocks, as shown in Fig. 4a. Probably, this is one of the reasons for the lower ductility of the samples partitioned at 350 and 450 °C. Besides being brittle, the local strain distribution in the vicinity of the fresh martensite reduced ductility and decreased the overall austenite stability as shown by De Knijf *et al*.^26^.

Figure 4b shows the tempered martensite microstructure in Q&P 450 sample. Average hardness reached about 470 HV in this sample. Carbon diffusion from martensite to the remaining austenite increases retained austenite stability^13,16,26^. The partitioning step at this temperature led to a coarsening of the cementite particles, thereby developing the film-like cementite in the microstructure, specially located at the grain boundaries. This situation is shown in Fig. 4b. It is known that cementite acts as brittle inclusions, accelerating the microcrack formation along boundaries by increasing stress concentration. Figure 4c depicts a complex microstructure containing tempered martensite with precipitated carbides, bainitic ferrite, and MA constituents. During the partition step, partial transformation of austenite to bainite containing precipitated carbides, as well as carbide free bainitic ferrite occurs. Zener^35^ proposed the incomplete reaction model, where bainitic ferrite can also be formed by partition of carbon atoms at the highest carbon concentration sites (i.e., at the grain boundaries). Nishikawa *et al*.^36^ showed that austenite carbon enrichment may occur not only due to partition from martensite, but also during bainitic reaction. Nevertheless, the austenite enrichment, if it occurred at all, was not enough to fully stabilize the remaining austenite. After the second quenching fresh martensite formed in the austenite regions forming MA microconstituent. Carbon partitioning from martensite to austenite grains, during the partitioning stage at 550 °C, contributes to a shift from a body-centered tetragonal (bct) distorted lattice to a more stable non-distorted body-centered cubic (bcc) lattice, accompanied by a decrease of the tetragonal distortion (c/a ratio). Therefore, lath martensite and a bainitic ferrite dispersion with about 385 ± 5 HV were developed in this sample.

As explained above, carbon solubility in martensite structure is much lower than in parent austenite. Then, trapped carbon atoms are continuously rejected when increasing the partitioning temperature. Transition carbides and cementite particles were nucleated where the carbon concentration reached its critical amount, consequently the carbide will be present as discontinuous stringers and isolated particles along the lath boundaries. Finally, the fragmented cementite lamellae morphology becomes more continuous when partitioning at at 650 °C^37^. On some of larger blocky austenite chunks a cooperative eutectoid decomposition took place, creating faceted regular pearlite grains, as can be seen seen in Fig. 4d (see inset). The partitioning step at 650 °C resulted on a hardness reduction to 340 ± 5 HV.

**Figure 5:**
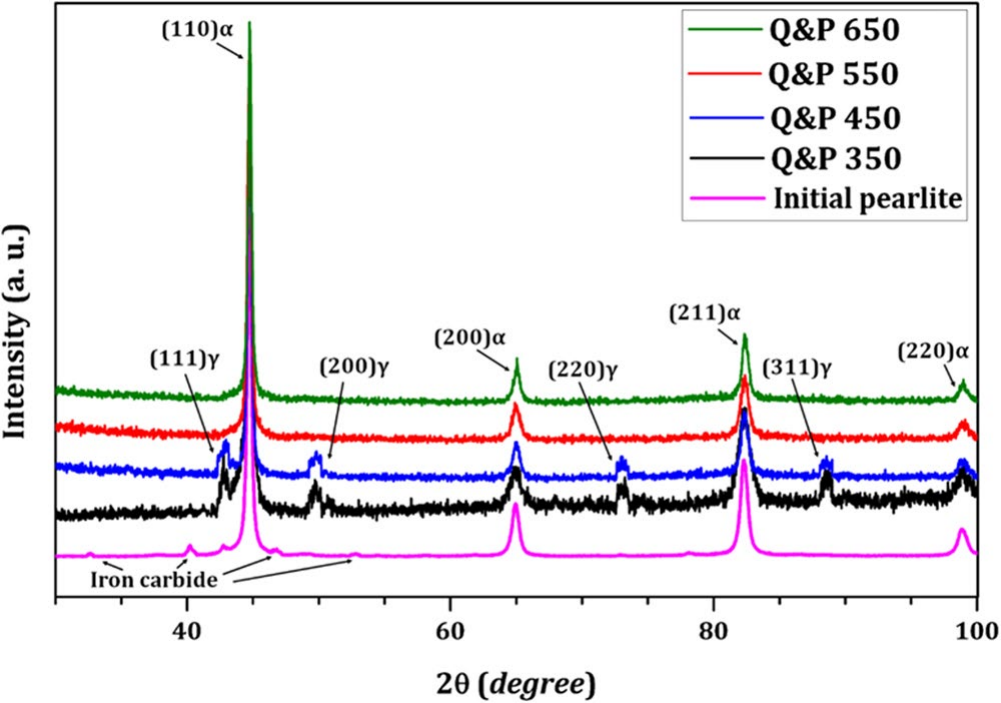
X–ray diffraction patterns of investigated specimens.

Figure 5 shows the XRD patterns of initial pearlite and the quench-partitioned samples. XRD results of the initial state revealed the dominant BCC-ferrite as the matrix of the pearlite structure with a small amount of carbides as products of eutectoid transformation. The results also demonstrated that the iron carbides (i.e., cementite) were fully dissolved in the matrix by austenitization at 900 °C. Moreover, no cementite or carbides peaks were found in Q&P 350 and 450 samples. The results show a significant difference between the investigated treatment (Q&P) and the traditional tempering, where fine cementite is developed during the second stage of aging of medium and high carbon content steels (greater than 350 °C)^38^. The determination of the retained austenite fraction and its stability at room temperature is crucial to predict mechanical performance.

X-ray diffraction analysis is a reliable method for determining austenite volume fraction higher than 3%^39,40^. XRD pattern for Q&P 350 and 450 samples revealed the presence of retained austenite at about 13 and 9% volume fraction, respectively. Although the retained austenite can contribute to the improvement of mechanical and wear behaviour by transformation induced plasticity phenomenon, the presence of brittle fresh martensite in Q&P 350 sample certainly reduces the toughness and plasticity, enhancing the brittle behaviour. Dislocation density can also be estimated using a numerical (i.e., Lorentzian and Gaussian) method from the broadening of X-ray diffraction peaks^39,40^. The calculated dislocation densities were 1.19 × 10^16^, 9.92 × 10^15^, 1.92 × 10^15^, and 8.04 × 10^14^ m^−2^ for Q&P 350, 450, 550, and 650 °C, respectively. The results showed the continuous reduction of dislocation density with increasing partitioning temperature as a consequence of martensite dislocations annihilation^41^.

**Figure 6:**
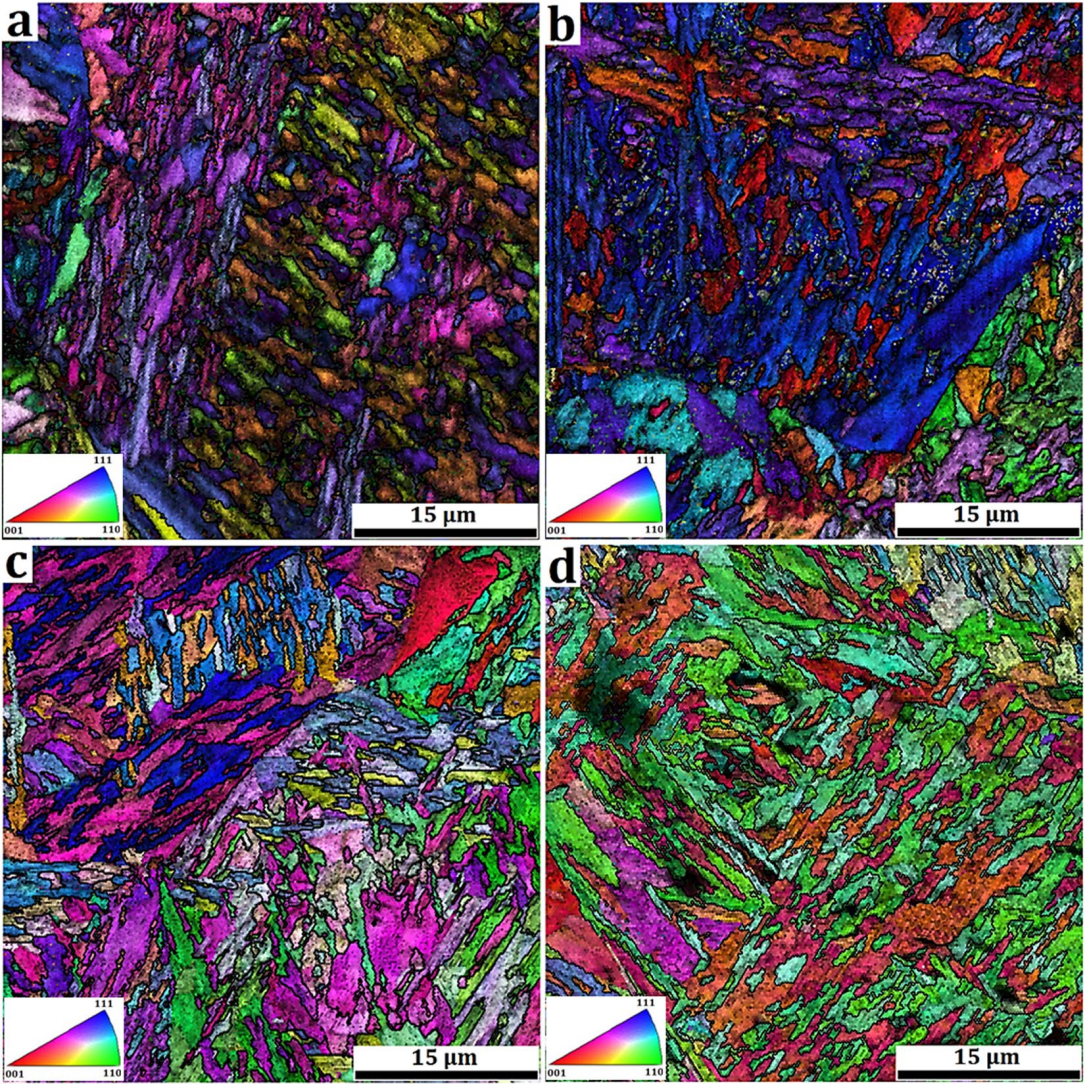
EBSD, orientation image maps (OIM) of (**a**) Q&P 350, (**b**) Q&P 450, (**c**) Q&P 550, and (**d**) Q&P 650 samples.

Figure 6 shows the EBSD orientation image maps (OIM) of treated samples. It was not possible to obtain good Kikuchi patterns quality from the carbides and cementite due to its discontinuities with high density of dislocations at interfaces. Therefore, the colors of OIMs indicate ferrite crystallographic orientations concerning to the pin axis in IPF (inverse pole figure) measurement. Martensite formed under rapid cooling from austenite region is accompanied by lattice distortion. In addition, BCC structure has notable elastic anisotropy. For instance, elastic modulus along <001> is much lower than along <110> and <111>^42^. Therefore, studying the variation of crystallographic orientation and the local strain gradient to the prediction of stress relief is crucial to the suggested treatment to be successful.

**Figure 7:**
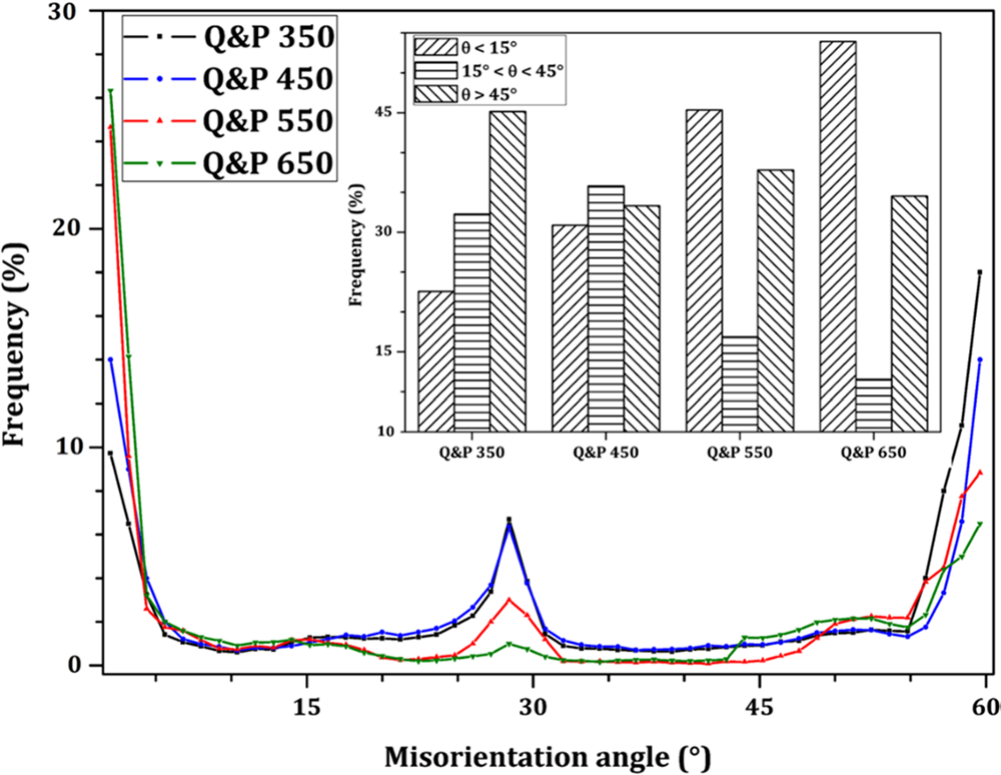
Comparison of point-to-point (relative) misorientation distribution of treated samples.

In general two types of boundaries can be discerned from the EBSD grain boundary maps: (i) subgrain or low angle boundaries (LABs, point-to-point misorientation less than 15°), and (ii) grain boundary or high angle grain boundaries (HABs, point-to-point misorientation greater than 15°). Figure 7 shows the comparison of point-to-point (relative) misorientation distribution of processed samples. Moreover, high angle boundaries have more mobility than low angle boundaries due to a higher density of dislocations and large stored energy^43^. Tsuchiyama *et al*.^44^ reported that the mobility of grain boundaries with misorientation angle in the range of 15–45° is high because it requires lower driving force energy for grain boundary migration. The distribution of boundary types for each sample is also displayed in Fig. 7. Q&P 350 and Q&P 450 samples exhibited the highest portion of HABs due to highest lattice distortion. On the other hand, Q&P 650 sample has the highest number of LABs or subgrains due to dislocation movement and annihilation (i.e., dynamic recovery) during tempering processing. In addition, the number of HABs with misorientation between 15–45° is higher in Q&P 350 and 450 samples, which is in line with Tsuchiyama work^44^. It means that a considerable number of mobile high angle boundaries exist in these samples due to incomplete dynamic recovery.

**Figure 8:**
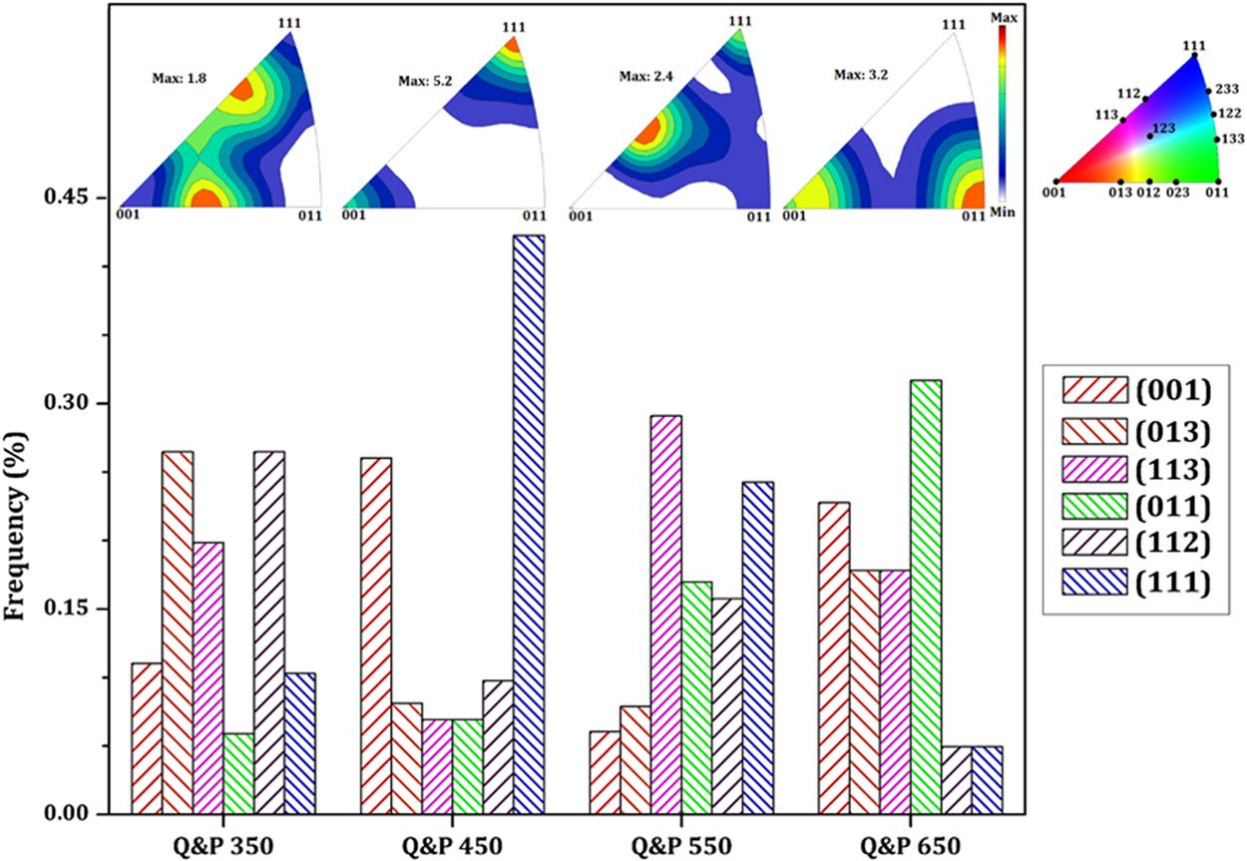
Distribution of crystallographic orientation calculated from related IPF.

The normal direction inverse pole figures (IPF) of each treated samples were calculated using statistical Kernel density estimation method^45,46^ and are presented in Fig. 8. Sainath *et al*.^47^ and Blondé *et al*.^48^ studied the orientation dependence of the BCC structure corresponding dislocation mobility. They ordered the gradual increase in elasticity modulus response by means of the orientation dependence from the interatomic distance of a specific plane as a function of dislocation theory (for instance, E_001_ ≪ E_011_ < E_112_ < E_111_). For better understanding, the normalized fraction of mean planes is also shown in Fig. 8. In the Q&P 550 sample, one can find a combination of significant amounts of crystals aligned to compact atomic planes, such as (110), (112), and (123), which have accessible slip systems aligned to the load direction. Therefore, it is expected that the mentioned sample will exhibit superior mechanical properties. Conversely, a considerable amount of (001) and (013) crystals increases the possibility of early fracture in Q&P 350 and Q&P 450 samples.

**Figure 9:**
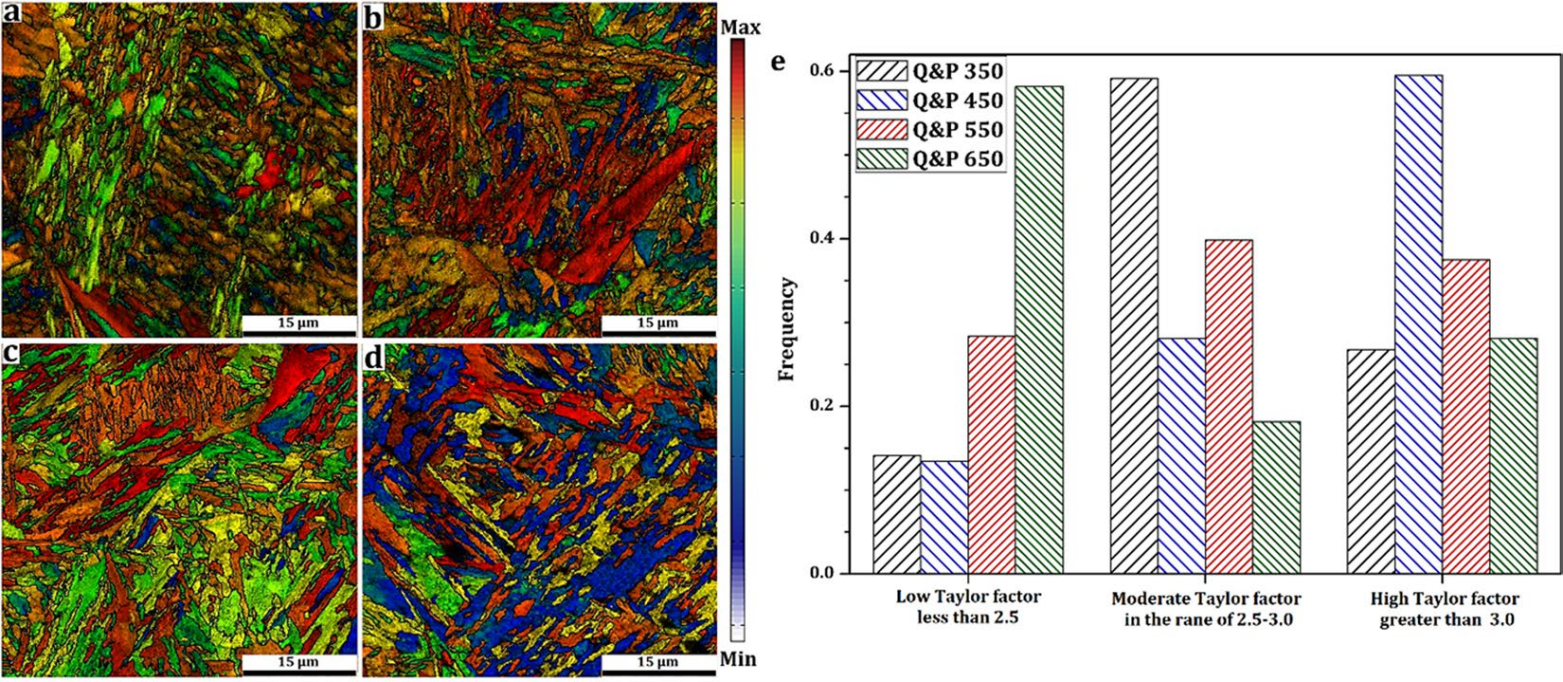
Taylor factor maps and comparison between low, moderate and high Taylor factor of treated samples.

Dislocation movement and their interaction (e.g., dislocation multiplication and annihilation) during the partitioning stage play a significant role in mechanical performance by releasing the stored energy in crystalline defects. Taylor factor can be used to determine the probability of dislocation movement occurring by the relief-stored energy. In BCC structures, Taylor factor values correspond to the minimum lattice rotations for an specific grain in arbitrary planes (pencil-glide dislocation slip in any {110}, {112}, or {123} planes but only along <111> direction)^49^. Taylor factor maps and comparisons among low, moderate, and high Taylor factors of each sample are presented in Fig. 9. In the low Taylor factor grains, their slip planes are already oriented along stored strain energy; therefore, minimum resolved shear stress for dislocation movement is needed. The Moderate Taylor factor grains easily achieved the critical resolved shear stress with modest grain rotation. While in high Taylor factor grains or hard grains the slip planes cannot be achieved by rotation^50,51^. The results showed that the lowest distribution of Taylor factors was obtained in Q&P 650 sample due to the dominance of {011} planes. In this case, the close-compact plane in BCC structure provides adequate slip systems for dislocation movement. However, the highest Taylor factor values are found in Q&P 350 and Q&P 450 samples due to the high dislocation densities and dislocation interaction (probably because recovery was not completed). Moreover, a considerable amount of {001} crystals with the highest interatomic distance may have prevented the dislocation slip. In other words, the creation of dislocation accumulation and dislocation walls could be favored at high Taylor factor grains, leading to the formation of microcracks due to local stress gradient. Therefore, inferior mechanical properties and early fracture are expected for Q&P 350 and 450 samples.

**Figure 10:**
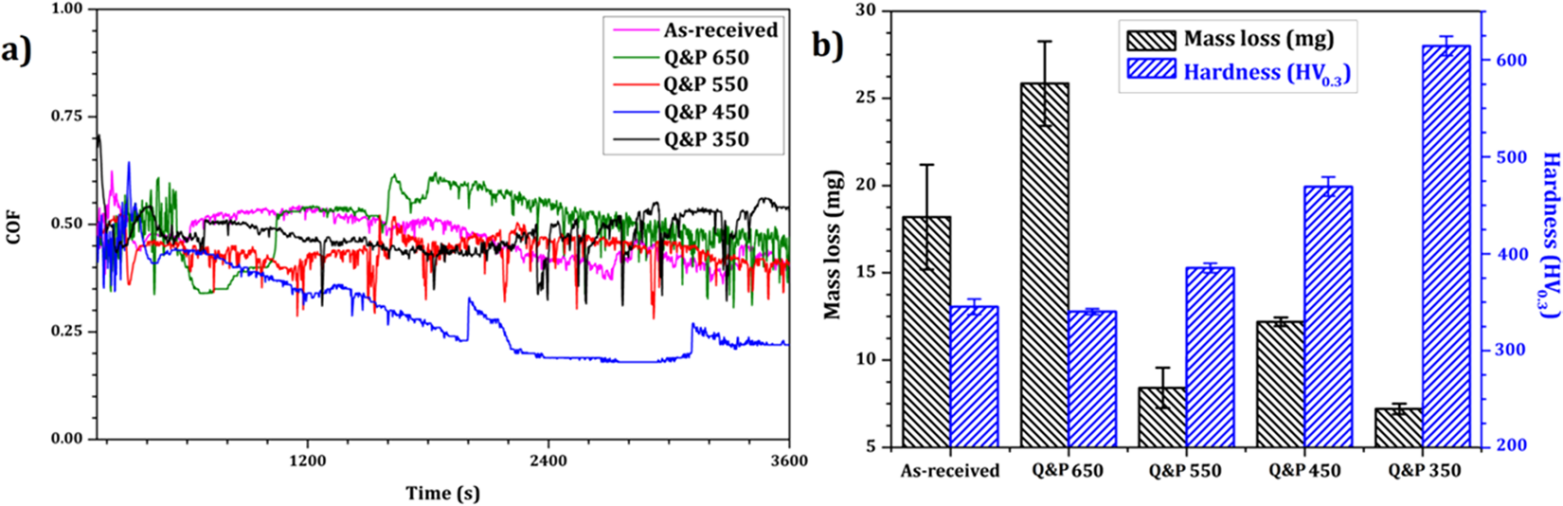
Variation of: (**a**) friction coefficient as a function of time, (**b**) mass loss and pin hardness of each sample.

Sliding wear tests were carried out on as received pearlitic material and on Q&P treated samples using a pin-on-disc wear machine. The variation of the friction coefficient of all tests is plotted in Fig. 10a. Two distinct modes of wear were identified, a running-in and a steady state. During the running-in stage, the asperities on the pin contact surface produce a considerable amount of wear. After a short time (about 60 s), the contact rubbing surfaces (pin and disc contact surface) were mated, and more real contact areas were achieved, causing a reduction of wear and changing the wear mode to steady state^23,24,52^. Q&P 550 pin sample exhibited the lowest wear coefficient among the samples that were investigated in the tribological system. Figure 10b shows the mass loss as a function of the pin hardness. The lowest mass loss was found in Q&P 350 samples, and it can be associated with higher brittle fresh martensite formation in the microstructure. However, it led to the highest coefficient of friction (COF) and an increase in the formation of undesirable rolling contact fatigue cracks. From the pin-on-disc tests results, Q&P 450 and 550 samples exhibited the best combination of hardness, wear coefficient, and mass loss in comparison with initial pearlite structure. Consequently, the results demonstrated that the Q&P process proposed for samples Q&P 550 may be applied for railway systems.

**Figure 11:**
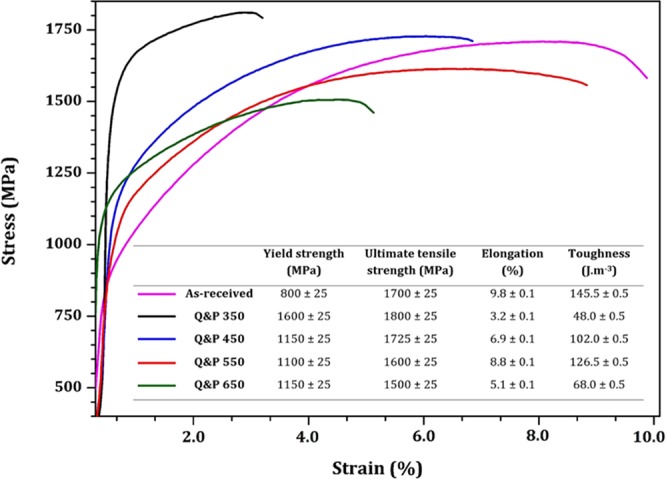
Stress-strain curves and mechanical properties of investigated specimens.

Furthermore, to explore the effect of the quenching and partitioning treatments, all investigated samples were uniaxial tensile tested. The stress-strain curves are presented in Fig. 11. The yield stress, the ultimate tensile strength, total elongation, and the toughness (area under stress-strain curves) were also calculated and listed in Fig. 11. The initial pearlite rail exhibited limited plastic strain and broke at an elongation lower than 10%. It may be associated with the head hardening treatment during rail processing to increase the wear resistance. Mädler *et al*.^53^ reported that for extra heavy loading service the minimum tensile strength of 1200 MPa is required to achieve the excellent wear resistance. All Q&P treated samples successfully reached this requirement; however, Q&P 350 sample, with higher yield strength, exhibited very early brittle fracture. This can be explained by the absence of stress relief during the tempering stage and the formation of brittle fresh martensite in its microstructure. The optimized tensile properties were achieved in Q&P 450 and 550 samples, which met the requirement of European standard (BS EN 13674 - Railway applications. Track. Rail). The reduction of the stored internal energy, the formation of tempered martensite and bainitic ferrite during the partitioning step, and the dominance of planes with enough slip systems enhanced the tensile behavior. Finally, the tensile mechanical properties in Q&P 650 significantly decreased due to the nucleation of pearlite colonies in the microstructure. Embrittlement phenomenon took place in this sample, which might be associated with the dispersion of fine cementite particles. The formation and growth of cementite generate compressive residual stresses in the ferrite and may form narrow bands of locally intense shear stress^54^, which can explain the reduction of tensile properties and wear resistance in Q&P 650 sample.

**Figure 12:**
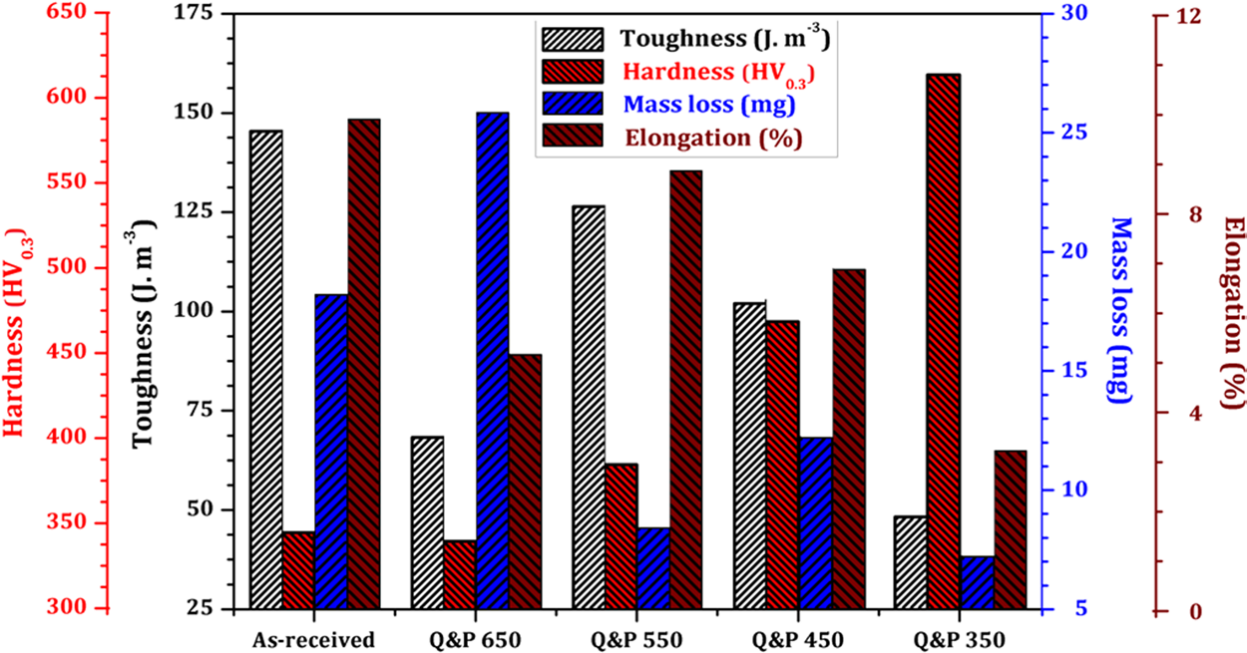
Comparison of wear resistance and tensile properties as a function of hardness of the investigated specimens.

The variation of wear resistance and tensile properties as a function of hardness of investigated specimens is summarized in Fig. 12. It is well known that wear resistance gradually increases with hardness. However, the lowest tensile mechanical properties and wear resistance is found in Q&P 350 sample, which presented the highest hardness caused by the formation of brittle fresh martensite and the most considerable residual stress induced by the martensitic transformation. The Q&P 650 sample contains a considerable amount of pearlite islands and the as-received is fully pearlitic, therefore, both exhibited higher mass loss. Finally, the best combination of hardness, wear resistance, and tensile properties were found in Q&P 450 and 550 samples, which can be associated with the tempered martensite and bainitic-ferrite present in their microstructure. Moreover, the presence of 9% and 13% of retained austenite in Q&P 450 and Q&P 550 samples resulted in the highest formability index^17^ (absorbed energy) by transformation-induced plasticity (TRIP) effect, which are unavoidable conditions of the railroad system. In addition, the effect of crystallographic textures on microstructure and mechanical properties were studied using EBSD technique. EBSD results predicted the presence of crystals along the close-compact planes which enhanced dislocation movement in these samples (Q&P 450 and Q&P 550), consequently avoiding the formation of dislocation wall structure and micro-cracks.

## Conclusions

This study investigated the novel heat treatment called quenching and partitioning processing to develop new microstructural features with the aim of increasing the wear resistance of a hypereutectoid steel, which is of great interest for heavy-haul railway service. The following conclusions were obtained:

- Complex microstructures containing tempered martensite, bainitic-ferrite, pearlite colonies, and retained austenite can be formed by controlling quenching and partitioning temperatures.
- The best combination of hardness, wear resistance, and tensile mechanical properties were found in samples quenched at 140 °C and partitioned at 450 and 550 °C, because of the formation of tempered martensite and bainitic-ferrite in the microstructure.
- X-ray diffraction analysis demonstrated the presence of 13 and 9% of retained austenite in the samples partitioned at 350 and 450 °C, respectively, which contributed to enhance the ductility by the TRIP effect.
- A continuous decrease in dislocation density was observed as a function of partitioning temperature.
- A considerable number of mobile boundaries (15° < θ < 45°) in samples partitioned at 350 and 450 °C can be related to incomplete dynamic recovery.
- Superior wear resistance can be obtained for samples quenched at 140 °C and then partitioned at 550 °C when compared to the pure/commercial pearlitic microstructure.
- Significant lowering of the elongation was found in the sample partitioned at 650 °C, which might be associated with the coarsening of cementite particles.
- The investigation of crystallographic orientation and the Taylor factor analysis from EBSD data successfully predicted the mechanical behavior.
